# Moderating Effects of Individual Characteristics and the Target Lower Limb Muscle Group on Flexibility Adaptations to Chronic Static Stretching in Healthy Individuals: A Systematic Review and Meta-Analysis of Randomized Controlled Trials

**DOI:** 10.1186/s40798-026-01066-1

**Published:** 2026-07-11

**Authors:** Kensuke Oba, Shingo Matsuo, Masatoshi Nakamura, Gakuto Nakao, Taizan Fukaya, Takamasa Mizuno, Kosuke Takeuchi

**Affiliations:** 1 Center for Environmental and Health Sciences, Hokkaido University, Kita 12, Nishi 7, Kita-Ku, Sapporo, Hokkaido 060-0812 Japan; 2 Department of Rehabilitation, Faculty of Health Sciences, Nihon Fukushi University, Handa, Aichi Japan; 3 Department of Physical Therapy, Faculty of Rehabilitation Sciences, Nishi Kyushu University, Kanzaki-Cho, Saga Japan; 4 SPLYZA Inc, Hamamatsu, Shizuoka Japan; 5 Department of Physical Therapy, School of Health Sciences, Sapporo Medical University, Sapporo, Hokkaido Japan; 6 Department of Physical Therapy, Faculty of Social Work Studies, Josai International University, Togane, Chiba Japan; 7 Research Center of Health, Physical Fitness and Sports, Nagoya University, Nagoya, Aichi Japan; 8 Department of Physical Therapy, Faculty of Rehabilitation, Kobe International University, Kobe, Hyogo Japan

**Keywords:** Range of motion, Stretch training, Joint mobility, Chronic effect

## Abstract

**Background:**

Static stretching is widely accessible across the lifespan and commonly prescribed to improve flexibility. However, whether flexibility improvements following chronic static stretching are moderated by participant characteristics or the targeted muscle group in lower-limb remains uncertain.

**Objectives:**

We primarily aimed to determine whether the effects of chronic static stretching on flexibility are moderated by age, sex, training status, baseline flexibility, and muscle group.

**Methods:**

We searched PubMed, Web of Science, and Scopus for randomized controlled trials published before June 2025 that examined the chronic effects of static stretching on lower limb flexibility outcomes (compared with non-stretching or passive control conditions) in healthy populations. Meta-analysis was performed using a random-effects model, with effect sizes expressed as standardized mean differences corrected for small-sample bias (Hedges’ g). Subgroup analyses were conducted to examine the potential moderation by age (children and adolescents vs young vs middle-aged vs older adults), sex (male vs female), training status (athlete/trained vs recreationally active vs sedentary), baseline flexibility (with tightness vs without tightness and not reported), and muscle group (knee flexors vs knee extensors vs ankle plantar flexors). Methodological quality was assessed using the Physiotherapy Evidence Database (PEDro) scale, and potential small-study effects were evaluated using Egger’s regression test and trim-and-fill analysis.

**Results:**

Seventy-nine studies (n = 3287) were included in the analysis. Chronic static stretching produced a moderate improvement in flexibility (Hedges’ g = 0.851, 95% confidence interval 0.710 to 0.991; SE = 0.0718; Z = 11.845; *p* < 0.001), with substantial heterogeneity (τ^2^ = 0.249; I^2^ = 66.1%). No significant moderating effects were observed for age, sex, training status, baseline flexibility, or target muscle group. Egger’s regression test indicated potential small-study effects (*p* < 0.001); however, trim-and-fill analysis did not impute any potentially missing studies and the adjusted pooled estimate remained unchanged. The mean PEDro score was 6.15 ± 0.90 (range 4–8), indicating moderate-to-good study quality.

**Conclusions:**

Chronic static stretching yielded significant, moderate improvements in lower-limb flexibility; however, substantial heterogeneity and potential small-study effects were observed. These effects did not appear to be meaningfully moderated by age, sex, baseline flexibility, training status, or target muscle group. Overall, chronic static stretching may improve lower-limb flexibility across diverse healthy individuals and target muscle groups, although the results should be interpreted cautiously given the heterogeneity, potential small-study effects, and methodological variability among the included studies.

**Supplementary Information:**

The online version contains supplementary material available at 10.1186/s40798-026-01066-1.

## Key Points

**Table:** 

Evidence from randomized controlled trials suggests that chronic static stretching is associated with a moderate improvement in lower-limb flexibility among healthy individuals, although substantial heterogeneity and potential small-study effects should be considered.
Age, sex, training status, baseline flexibility, and target muscle group did not appear to meaningfully moderate the flexibility improvements associated with chronic static stretching.
Chronic static stretching may improve lower-limb flexibility across different target muscle groups, including the knee flexors, knee extensors, and ankle plantar flexors, although the magnitude of improvement may vary numerically across studies.

## Introduction

Static stretching involves holding a joint position that elongates soft tissues beyond slack length until passive resistance, stretch sensation, or discomfort is experienced, with the aim of increasing joint range of motion (ROM) [1, 2]. In addition to enhancing ROM, static stretching can reduce muscle stiffness [3]. Its role in injury prevention remains controversial; a recent systematic review and meta-analysis suggested that static stretching interventions may reduce the risk of muscle injuries, but not tendon injuries, in healthy active individuals [4]. Owing to its simplicity and minimal physical demands, static stretching is widely accessible across the lifespan and routinely incorporated into physical education, rehabilitation, athletic training, and general fitness programs. Given its extensive use in both clinical and athletic contexts, identifying the static stretching variables that optimize ROM enhancement—including stretch duration, intensity, and frequency—and examining how individual characteristics (e.g., age, sex, baseline flexibility, and training status) influence these adaptations are essential.

Recent systematic reviews and meta-analyses [5–8] have reported moderate-to-large effects of chronic static stretching on ROM. These reviews have primarily focused on the training variables that modulate static stretch-induced adaptations. Arntz et al. [7] demonstrated a positive dose–response relationship between ROM improvements and stretch volume (indexed by a greater number of sets per session, longer session duration, and higher total stretching time) using a meta-regression analysis. Similarly, Ingram et al. [5], in an exploratory multivariate meta-regression, reported that ROM improvements after static stretching program plateaued at approximately 4 min/session or 10 min/week, with no additional benefits observed beyond these thresholds. Notably, stretch intensity was not consistently associated with ROM improvements across recent meta-analyses [5–7], implying that the cumulative stretching stimulus should be prioritized. Collectively, this body of evidence identifies total stretch volume as the principal determinant of flexibility adaptations and delineates specific quantitative thresholds for optimizing outcomes [5].

Although training variables have been systematically investigated, the role of individual characteristics in moderating stretching-induced adaptations remains poorly understood. Konrad et al. [6] suggested that women may experience greater ROM improvements than men and that baseline training status does not substantially influence flexibility outcomes. However, their meta-analysis pooled multiple stretching modalities (dynamic, ballistic, and proprioceptive neuromuscular facilitation (PNF) stretching), and the findings cannot be directly attributed to static stretching. Conversely, Ingram et al. [5], who focused exclusively on static stretching interventions, included participants with heterogeneous health profiles and restricted their sample to adults (i.e., ≥ 18 years). However, the inclusion of non-healthy participants may elicit static stretching responses that differ from those observed in healthy individuals, thereby constraining external validity and limiting the generalizability of the conclusions. Furthermore, several recent systematic reviews and meta-analyses have incorporated non-randomized trials, thereby raising concerns regarding heterogeneity in baseline characteristics and the potential influence of selection bias on the reliability of their findings [5–8]. Moreover, a narrative review [9] indicated that static stretching–induced changes in muscle mechanical properties may vary among target muscle groups, highlighting the need to clarify whether such responses differ by muscle group.

To address this gap in the knowledge, we conducted a systematic review and meta-analysis with the primary aim of determining the chronic effects of static stretching on flexibility in healthy individuals. The secondary aim was to assess whether these effects vary according to individual characteristics (e.g., sex, age, training status, and baseline flexibility) and the target muscle group. To address these aims, three key design features were adopted. First, only randomized controlled trials (RCTs) were included to maximize internal validity and minimize selection bias. Second, participants across the entire lifespan—from children to older adults—were included to enable subgroup analyses by age, as flexibility is relevant to motor development in youth, performance in adulthood, and mobility and fall prevention in older age. Third, the analysis was limited to lower-limb muscles and joints to define a focused and clinically relevant scope, given the importance of lower-limb flexibility for locomotion, athletic performance, and musculoskeletal injury. Therefore, the present findings should be interpreted as evidence specific to lower-limb flexibility adaptations and not generalized to upper-limb muscles. These data may help determine whether recommendations for static stretching to increase flexibility should account for individual characteristics.

## Methods

This systematic review and meta-analysis were conducted in accordance with the Preferred Reporting Items for Systematic Reviews and Meta-Analyses statement 2020 [10].

### Information Sources and Search Strategy

Electronic literature searches were conducted in PubMed, Web of Science, and Scopus. The search was performed on June 24, 2025, and all studies published prior to that date were included in the study. The following search strategy was applied uniformly across all three databases: (flexibility[Title/Abstract] OR “range of motion”[Title/Abstract] OR extensibility[Title/Abstract] OR “joint mobility”[Title/Abstract]) AND (stretch*[Title/Abstract]) AND (“randomized controlled trial”[Publication Type] OR “controlled clinical trial”[Publication Type] OR randomized[Title/Abstract] OR randomised[Title/Abstract] OR randomly[Title/Abstract] OR trial[Title/Abstract]).

### Inclusion and Exclusion Criteria

Articles were included in this systematic review and meta-analysis if they met the following criteria:

*Population*: Studies involving individuals without musculoskeletal disorders, pain, or neurological impairments, regardless of age, sex, or training status.

*Intervention*: Static stretching exercises or training programs consisting of multiple sessions with an intervention duration ≥ 2 weeks [6]. Studies combining static stretching with other interventions (e.g., static stretching plus resistance training, proprioceptive neuromuscular facilitation, dynamic stretching, or ballistic stretching) were excluded. Studies in which participants performed the stretching intervention immediately prior to flexibility assessment were also excluded, as this could confound chronic adaptations with acute effects. To enhance homogeneity and external validity, interventions were restricted to lower limb muscle groups.

*Comparison*: A passive (non-stretching) control group in between-subject designs or the contralateral limb in within-subject designs [5].

*Outcome*: Objectively measured flexibility outcomes (e.g., ROM in degrees and distance in centimeters) were reported as pre- and post-intervention values, including the corresponding means and standard deviations (SDs).

*Study Design*: Eligible study designs included RCTs with baseline and follow-up assessments using either between-subject or within-subject designs. In within-subject designs, the contralateral limb served as the non-stretching control condition. Studies lacking pre- or post-intervention data were excluded. To minimize potential biases, such as participant selection bias and baseline imbalances, studies without a randomized or controlled intervention designs were excluded. Because within-subject contralateral-limb designs may be affected by crossover or remote effects, these studies were considered separately in a sensitivity analysis.

*Study Language, Publication Status, and Timefram*e: Only full-text, peer-reviewed journal articles published in English were considered, regardless of publication year. Review articles, case reports, special communications, letters to the editor, invited commentaries, and conference papers were excluded.

### Study Selection

All study selection procedures were performed by seven independent reviewers (KO, SM, MN, GN, TF, TM, and KT). Initially, the titles and abstracts of all retrieved records were independently screened by two reviewers from this group to assess eligibility. Studies that did not align with the aims of the study were excluded according to the predefined inclusion and exclusion criteria. Full-text articles were subsequently evaluated following this initial screening by two researchers (KO and SM). Any disagreements were resolved through consultation with a third researcher (KT).

### Data Extraction

The following data were extracted from each included study: (1) study characteristics, including the authors, year of publication, and sample size; (2) participant characteristics, such as sex, age, training status, and baseline flexibility; (3) details of the stretching intervention, including the stretching methods and specific muscle or muscle group targeted; and (4) flexibility measurements, including pre- and post-intervention values with corresponding SDs for both the stretching and control groups. When flexibility was assessed at multiple time points to evaluate prolonged effects, the value obtained closest to the end of the intervention period was selected for analysis. In studies targeting multiple target muscle groups but reporting limited outcome measures, the target muscle group was defined according to the reported flexibility outcome. Data extraction was performed independently by two reviewers, and any discrepancies were resolved through discussion until consensus was reached. When essential data were unavailable in the published reports, the corresponding authors were contacted via email or alternative platforms, such as ResearchGate. If no response was received, the study was excluded from the analysis.

### Statistics and Data Synthesis

Statistical analyses were conducted as previously described [6, 11, 12]. All meta-analyses were performed in R (version 4.5.2) using the metafor package. For each study, the mean change, SD of change, and sample size for the stretching and control conditions were extracted or calculated from the available data. When multiple measurements were reported within a single study, the mean changes and corresponding SDs were first combined within each group and subsequently used to calculate the study-level effect size. A random-effects model with restricted maximum likelihood estimation was used to calculate pooled effect sizes, expressed as standardized mean differences corrected for small-sample bias (Hedges’ g). Following the approach of previous meta-analyses [6, 11, 12], subgroup analyses were performed when at least three studies were available for a given subgroup. Consequently, subgroup analyses were conducted for age (children/adolescents [< 18 years], vs young adults [18–39 years], vs middle-aged adults [40–64 years], vs older adults [≥ 65 years]) [13, 14], sex (male vs female), training status (athlete and trained vs recreational and active vs sedentary), baseline flexibility (with tightness vs without tightness and not reported), and muscle group (knee flexor vs knee extensor vs ankle plantar flexor). The age categorization was selected to provide a more detailed assessment than the broad cut-off values (e.g., < 65 vs ≥ 65 years) or meta-regression approaches without predefined age group comparisons used in some previous meta-analyses [5, 7]. Training status was classified into athlete/trained, recreationally active, and sedentary based on study descriptions and guided by a previously proposed Tier 0–5 classification framework [15]. Participants were classified as athlete/trained, recreationally active, or sedentary only when these terms, or clearly equivalent descriptions, were reported in the original articles. Studies that did not provide sufficient information on training status were classified as “not reported” and excluded from the corresponding subgroup analysis. Baseline flexibility was classified as “with tightness” only when participants were explicitly described as having muscle tightness, limited flexibility, or restricted ROM at baseline. Studies without such explicit descriptions were classified as “without tightness/not reported” and were not considered as evidence of pre-existing tightness. Differences between subgroup effect sizes were evaluated using Q-statistics [6, 11, 12]. Effect sizes were interpreted as follows: < 0.2, trivial, 0.2–0.6, small; 0.6–1.2, moderate; 1.2–2.0, large; 2.0–4.0, very large; and > 4.0, extremely large [16]. Heterogeneity among studies was assessed using the I^2^ statistic and τ^2^. I^2^ thresholds of 25%, 50%, and 75% were interpreted as indicating low, moderate, and high heterogeneity, respectively [6, 11, 12]. Statistical significance was defined as a p-value of < 0.05 for all analyses.

### Risk of Bias Assessment and Methodological Quality

The methodological quality of the included studies was evaluated using the Physiotherapy Evidence Database (PEDro) scale [6, 11]. The PEDro scale consists of 11 methodological criteria, with each item scored as 0 or 1 by two independent assessors. Higher scores indicate better methodological quality. Discrepancies between the assessors’ scores were resolved through discussion to reach consensus.

### Publication Bias and Sensitivity Analyses

Potential small-study effects were assessed using Egger’s regression test. In addition, trim-and-fill analysis was performed using the trimfill function in the metafor package to evaluate the potential impact of small-study effects on the pooled effect estimate. Sensitivity analyses were performed after excluding studies using a within-subject contralateral-limb control design and, when applicable, studies with extremely large effect sizes according to the predefined interpretation threshold. These sensitivity analyses were conducted to examine whether the pooled estimate, heterogeneity, and potential small-study effects were materially influenced by study design or unusually large effects.

## Results

### Search Outcomes

**Fig. 1:**
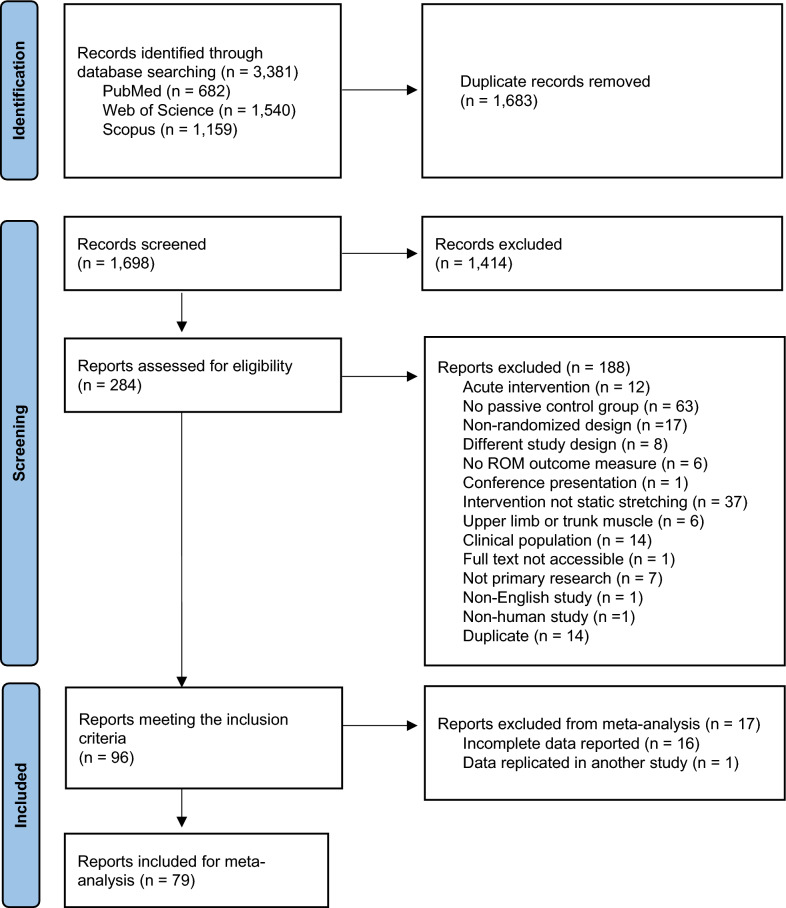
PRISMA flowchart of the study selection process

A total of 3381 records were identified through database searches after removing duplicates, 1698 records were screened, of which 96 met the eligibility criteria for inclusion. Of these, 17 studies were excluded because the necessary flexibility data could not be obtained from the corresponding authors or were duplicated in another study. Consequently, 79 studies were included in the final analysis [17–95]. Figure 1 presents the PRISMA flow diagram of the literature search and study selection process.

### Study Characteristics

The characteristics and primary outcomes of the included studies are summarized in Table 1. Among the 79 included studies, 74 employed a between-subject design (74/79 = 93.67%), whereas 5 used a contralateral extremity within-subject design (5/79 = 6.33%). With regard to age, 13 studies enrolled children or adolescents (< 18 years; 13/79 = 16.46%), 59 enrolled young adults (18–39 years; 59/79 = 74.68%), 3 enrolled middle-aged adults (40–64 years; 3/79 = 3.80%), and 4 enrolled older adults (≥ 65 years; 4/79 = 5.06%). In terms of sex, 20 studies included males participants only (20/79 = 25.32%), 9 included female participants only (9/79 = 11.39%), 49 included mixed-sex samples (49/79 = 62.03%), and 1 study did not report participant sex (1/79 = 1.27%). Training status could not be clearly classified in 48 studies owing to insufficient reporting (e.g., partial descriptions of exercise habits or vague categories such as “minimally active”); these studies were therefore excluded from this classification. Among the remaining 31 studies, 9 targeted athletes or strength-trained individuals (9/31= 29.03%); 19 targeted recreationally, physically, or generally active individuals (19/31 = 61.29%); and 3 targeted sedentary individuals (3/31 = 9.68%). With respect to baseline flexibility, 22 studies enrolled participants with predefined muscle tightness (22/79 = 27.85%), including knee flexor tightness in 18 studies, ankle plantar flexor tightness in 2 studies, knee extensor tightness in 1 study, and hip flexor tightness in 1 study. Among the remaining 57 studies (57/79 = 72.15%), participants were stratified into tight and non-tight groups at baseline in 1 study (1/79 = 1.27%), the absence of baseline tightness was confirmed in 4 studies (4/79 = 5.06%), and baseline tightness was not used for stratification but was considered in post-intervention grouping in 1 study (1/79 = 1.27%). Baseline flexibility was not reported in 51 studies (51/79 = 64.56%). With regard to the target muscle group, 47 studies focused on the knee flexors (47/79 = 59.49%), 22 on the ankle plantar flexors (22/79 = 27.85%), and 6 on the knee extensors (6/79 = 7.59%). One study each targeted the hip rotators (1/79 = 1.27%) and hip flexors (1/79 = 1.27%), whereas two studies involved a combination of multiple muscle groups and could not be assigned to a single target muscle group (2/79 = 2.53%).

**Table 1:** Summary of study characteristics and primary outcomes

Study	Study design	Sample size (n, men, women)^a^	Intervention group (n, men, women)	Control group (n)	Sex	Age	Participants’ activity/training status	Participants’ baseline flexibility	Target muscle group	Main outcome
Akagi and Takahashi 2014 [17]	RCT (Contralateral extremity in within-subject design)	19	19		Male	23.7 ± 2.3	Mixed Six participants were sedentary, and the others reported engaging in 1–8 h/week of recreational sports	Not reported	Ankle plantar flexors	Maximum ankle dorsiflexion angle (prone)
Andrade et al. 2020 [18]	RCT (Between-subject design)	60	21 (men = 10, women = 11)	17 (men = 9, women = 9)	Mixed	IG 21.0 ± 2.4 CG 21.1 ± 2.0	Not reported	Not reported	Ankle plantar flexors	Maximum ankle dorsiflexion angle
Aquino et al. 2010 [29]	RCT (Between-subject design)	45 (men = 6, women = 39)	15 (men = 2, women = 13)	15 (men = 2, women = 13)	Mixed	IG 22.6 ± 1.84 CG 22.33 ± 1.45	Not reported	Hamstring tightness (+)	Hamstring	Maximum knee extension angle at 110° hip flexion
Ayala et al. 2010 [51]	RCT (Between-subject design)	18	10	8	Female	21.3 ± 2.5	Athlete	Not reported	Hamstring	SLR angle
Ayala et al. 2010 [51]	RCT (Between-subject design)	150 (men = 106, women = 44)	IG1 35 (men = 22, women = 13) IG2 47 (men = 35, women = 12) IG3 39 (men = 27, women = 12)	29 (men = 22, women = 7)	Mixed	21.3 ± 2.5	Recreationally active	Hamstring tightness (-)	Hamstring	SLR angle
Ayala et al. 2013 [40]	RCT (Between-subject design)	161	IG1 39 (normal hamstring) IG2 32 (tight hamstring)	CG1 37 (normal hamstring) CG2 30 (tight hamstring)	Male	21.9 ± 1.7	Recreationally active	Grouping (1 vs 2) based on tightness	Hamstring	SLR angle
Bandy and Irion 1994 [94]	RCT (Between-subject design)	75 (men = 44, women = 31)	IG1 14 (men = 10, women = 4) IG2 14 (men = 10, women = 4) IG3 14 (men = 9, women = 5)	CG 15 (men = 11, women = 4)	Mixed	IG1 26.50 ± 4.69 IG2 24.64 ± 2.31 IG3 26.36 ± 6.66 CG 26.87 ± 6.42	Not reported	Hamstring tightness (+)	Hamstring	Passive knee extension test
Bandy et al. 1997 [84]	RCT (Between-subject design)	100	IG1 18 (men = 12, women = 6) IG2 19 (men = 12, women = 7) IG3 18 (men = 12, women = 6) IG4 18 (men = 12, women = 6)	CG 20 (men = 13, women = 7)	Mixed	IG1 24.44 ± 3.35 IG2 27.32 ± 5.6 IG3 27.33 ± 7.6 IG4 24.78 ± 2.37 CG 27.2 ± 4.79”	Not reported	Hamstring tightness (+)	Hamstring	Passive knee extension test
Bandy et al. 1998 [73]	RCT (Between-subject design)	58 (men = 41, women = 17)	19	20	Mixed	IG 24.63 ± 2.38 CG 28.35 ± 7.58	Not reported	Hamstring tightness (+)	Hamstring	Passive knee extension test
Barbosa et al. 2018 [95]	RCT (Between-subject design)	58	15	15	Male	IG 23.07 ± 3.5 CG 21.27 ± 2.8	Recreationally and physically active	Hamstring tightness (+)	Hamstring	Active knee extension test
Bazett-Jones et al. 2008 [19]	RCT (Between-subject design)	21	10	11	Female	18.57 ± 0.73	Athlete	Not reported	Hamstring	Active knee extension test
Ben and Harvey 2010 [20]	RCT (Between-subject design)	60 (men = 16, women = 44)	30 (men = 9, women = 21)	30 (men = 7, women = 23)	Mixed	IG 35 ± 12 CG 39 ± 12	Generally active	Hamstring tightness (-)	Hamstring	SLRangle
Blazevich et al. 2012 [21]	RCT (Between-subject design)	22	11	9	Male	18.7 ± 0.8	Not reported	Not reported	Ankle plantar flexors	Maximum ankle dorsiflexion angle
Borman et al. 2011 [22]	RCT (Between-subject design)	36 (men = 8, women = 28)	IG1 12 (men = 2, women = 10) IG2 12 (men = 4, women = 8)	CG 12 (men = 2, women = 10)	Mixed	43.6 ± 19.1	Not reported	Hamstring tightness (+)	Hamstring	Active knee extension test Lumbar ROM
Caldwell et al. 2019 [23]	RCT (Between-subject design)	30	IG1 10 (men = 4, women = 6) IG2 10 (men = 4, women = 6)	CG 10 (men = 4, women = 6)	Mixed	IG1 22.6 ± 2.7 IG2 22.6 ± 2.7 CG 21.4 ± 3.8	Recreationally active	Not reported	Hamstring Quadriceps	SLR angle
Castro et al. 2013 [33]	RCT (Between-subject design)	162	63	99	Male	13.3 ± 2.7	Athlete	Not reported	Hip rotators	Hip internal rotation ROM Hip external rotation ROM Total hip rotation ROM (right) Total hip rotation ROM (left) Average of right and left total hip rotation ROM
Chan et al. 2001 [24]	RCT (Between-subject design)	40 (men = 24, women = 16)	IG1 10 (men = 6, women = 4) IG2 10 (men = 6, women = 4)	CG1 10 (men = 6, women = 4) CG2 10 (men = 6, women = 4)	Mixed	IG1 21.7 ± 3.6 CG1 20.6 ± 2.5 IG2 19.3 ± 0.7 CG2 20.1 ± 2.2	Not reported	Not reported	Hamstring	Passive knee extension test
Chaudhuri and Kiba 2018 [25]	RCT (Between-subject design)	50 (men = 26, female = 24)	IG1 10 (men = 6, women = 4) IG2 10 (men = 6, women = 4) IG3 10 (men = 4, women = 6) IG4 10 (men = 4, women = 6)	CG 10 (men = 6, female = 4)	Mixed	IG1 24.7 ± 2.6 IG2 24.3 ± 3.12 IG3 24.4 ± 2.79 IG4 24.4 ± 3.27 CG 24.5 ± 2.87	Not reported	Rectus femoris tightness (+)	Quadriceps	Active knee flexion angle (prone)
Christiansen 2008 [26]	RCT (Between-subject design)	40	18 (men = 3, women = 15)	19 (men = 5, women = 14)	Mixed	IG 72.5 ± 4.7 CG 71.6 ± 5.0	Not reported	Not reported	Ankle plantar flexors Hip flexors	Maximum ankle dorsiflexion angle Hip extension angle in the Thomas test position
Cini et al. 2020 [27]	RCT (Between-subject design)	18	6	6	Female	IG 24 ± 2.8 CG 24.6 ± 2.6”	Sedentary or insufficiently active	Not reported	Hamstring	SLR angle
Cini et al. 2024 [28]	RCT (Between-subject design)	30	IG1 10 (men = 1, women = 9) IG2 10 (men = 2, women = 8)	10 (men = 3, women = 7)	Mixed	IG1 22.6 ± 3.02 IG2 24.9 ± 6.44 CG 23.8 ± 3.73	Physically active	Not reported	Ankle plantar flexors	Maximum ankle dorsiflexion angle Ankle Lunge Test
Cipriani et al. 2012 [30]	RCT (Between-subject design)	62	IG1 11 IG2 12 IG3 11 IG4 10	9	Mixed	24 ± 5.5	Not reported (non-athlete)	Hamstring tightness (-)	Hamstring	SLR angle
Covert et al. 2010 [31]	RCT (Between-subject design)	62	11 (men = 4, women = 7)	11 (men = 6, women = 5)	Mixed	IG 21.82 ± 2.14 CG 21.45 ± 3.06	Not reported	Hamstring tightness (+)	Hamstring	Passive knee extension test
Dal Paz et al. 2018 [32]	RCT (Between-subject design)	38	10	8	Female	IG 66 ± 8 CG 68 ± 5	Sedentary	Not reported	Hamstring	SLR angle
Fernandez et al. 2016 [76]	RCT (Between-subject design)	103	81	22	Male	IG 18.9 ± 1.8 CG 16.5 ± 0.7	Athlete	Not reported	Hamstring	Sit and reach test
Folpp et al. 2006 [35]	RCT (contralateral extremity in within-subject design)	20 (men = 8, women = 12)	20 (men = 8, women = 12)		Mixed	24 ± 6.5	Not reported	Hamstring tightness (+)	Hamstring	SLR angle
Gajdosik et al. 2005 [37]	RCT (Between-subject design)	19	10	9	Female	IG 73.1 ± 6.8 CG 75.3 ± 8.3	Minimally to moderately active	Ankle plantar flexors tightness (+)	Ankle plantar flexors	Maximum ankle dorsiflexion angle Total dorsiflexion and plantarflexion ROM
Gajdosik et al. 2007 [36]	RCT (Between-subject design)	12	6	4	Female	IG 23 ± 4 CG 21 ± 1	Sedentary	Not reported	Ankle plantar flexors	Maximum ankle dorsiflexion angle Total dorsiflexion and plantarflexion ROM
Gallo et al. 2015 [88]	RCT (Between-subject design)	31	13	13	Female	IG 69.9 ± 8.6 CG 66.6 ± 6.0	Not reported	Not reported	Hamstrings Ankle plantar flexors Quadriceps Hip flexors Hip extensors Shoulder flexor Elbow flexor	Sit and reach test
Godges et al. 1993 [38]	RCT (Between-subject design)	25	9	7	Male	21 ± 1	Recreational athlete	Hip flexors tightness (+)	Hip flexors	Hip extension ROM measured using the modified Thomas test
Gribble et al. 1999 [39]	RCT (Between-subject design)	45	12	16	Mixed	19.67 ± 1.55	Not reported	Not reported	Hamstring	SLR Active knee extension test
Gunaydin et al. 2020 [41]	RCT (Between-subject design)	56 (men = 31, female = 25)	14	14	Mixed	IG 24.07 ± 3.2 CG 22.43 ± 2.62	Not reported	Not reported	Hamstring	Active knee extension test
Henderson et al. 2025 [42]	RCT (Between-subject design)	44 (men = 20, women = 24)	14 (men = 8, women = 6)	14 (men = 7, women = 7)	Mixed	IG 23.5 ± 4.3 CG 24.5 ± 3.6	Mixed Exercise 1–2 × /week: IG 71%, CG 64%	Hamstring tightness (+)	Hamstring	Passive knee extension test
Ikeda and Tomoo 2021 [43]	RCT (Between-subject design)	25	12	13	Male	IG 22 ± 1 CG 22 ± 2	Mixed Six participants (total) engaged in recreational sports 1–2 × /week (mostly ball games); others reported not participating in regular sports	Not reported	Quadriceps	Maximum knee flexion angle (prone)
Johanson et al. 2009 [44]	RCT (Between-subject design)	16 (men = 9, women = 7)	8 (men = 4, women = 4)	8 (men = 5, women = 3)	Mixed	IG 29.0 ± 10.8 CG 25.9 ± 5.5	Not reported	Not reported	Ankle plantar flexors	Maximum ankle dorsiflexion angle
Johnson et al. 2014 [45]	RCT (Between-subject design)	49	14	8	Not reported	22 ± 4.4	Not reported	Hamstring tightness (+)	Hamstring	Passive knee extension test
Junpei et al. 2023 [46]	RCT (Between-subject design)	18	9	9	Male	IG 21.9 ± 0.7 CG 22.1 ± 1.5	Not reported	Not reported	Ankle plantar flexors	Maximum knee flexion angle
Kim et al. 2017 [47]	RCT (Between-subject design)	29 (men = 25, women = 4)	7	7	Mixed	29.2 ± 5.8	Not reported	Hamstring tightness (+)	Hamstring	Finger-to-floor test Passive knee extension test
Knight et al. 2001 [48]	RCT (Between-subject design)	97 (men = 38, women = 59)	19 (men = 8, women = 11)	18 (men = 5, women = 13)	Mixed	IG 27.37 CG 26.17 SD not reported	Not reported	Ankle plantar flexors tightness (+)	Ankle plantar flexors	Maximum ankle dorsiflexion angle (knee flexed and extended)
Kokkonen et al. 2007 [49]	RCT (Between-subject design)	38 (men = 16, women = 22)	19 (men = 8, women = 11)	19 (men = 8, women = 11)	Mixed	IG(male) 23 ± 3 IG(female) 22 ± 4 CG(male) 25 ± 1 CG(female) 22 ± 4	Inactive or recreationally active	Not reported	Major lower-extremity muscle groups (hamstrings, quadriceps, hip adductors, hip abductors, hip external/internal rotators, plantar flexors, and dorsiflexors)	Sit and reach test
Konrad and Tilp 2014 [50]	RCT (Between-subject design)	49 (men = 35, women = 14)	20	18	Mixed	Male 23.3 ± 2.9 Female 22.5 ± 2.5	Not reported	Not reported	Ankle plantar flexors	Maximum ankle dorsiflexion angle
Laudner et al. 2016 [52]	RCT (Between-subject design)	34 (men = 15, women = 19)	IG1 12 IG2 12	10	Mixed	IG1 21.3 ± 1.6 IG2 21 ± 1.5 CG 22.2 ± 2.3	Physically active	Not reported	Hamstring	SLR angle
Li et al. 1996 [53]	RCT (Between-subject design)	39 (men = 17, women = 22)	14 (men = 8, women = 11)	15 (men = 9, women = 11)	Mixed	IG 28.4 ± 5.1 CG 29.3 ± 3.5	Not reported	Hamstring tightness (+)	Hamstring	SLR angle Active knee extension test
Lima et al. 2015 [34]	RCT (Between-subject design)	24	12	11	Male	19.05 ± 1.4	Physically active male	Not reported	Quadriceps Hamstring	Maximum knee flexion angle (prone) Passive knee extension test
Longo et al. 2021 [54]	RCT (Between-subject design)	30 (men = 18, women = 12)	15 (men = 9, women = 6)	15 (men = 9, women = 6)	Mixed	IG 22.3 ± 0.8 CG 23.4 ± 0.8	Recreationally active	Not reported	Ankle plantar flexors	Maximum knee flexion angle (prone)
Mahieu et al. 2007 [55]	RCT (Between-subject design)	96	31 (men = 21, women = 10)	29 (men = 8, women = 21)	Mixed	IG 22.03 ± 1.11 CG22.31 ± 1.91	Recreational athlete	Not reported	Ankle plantar flexors	Maximum ankle dorsiflexion angle
Maras et al. 2024 [56]	RCT (Between-subject design)	52	15	15	Mixed	IG 24.26 ± 3.82 CG 28.73 ± 5.95	Not reported	Hamstring tightness (+)	Hamstring	Active knee extension test Sit and reach test SLR angle
Marshall et al. 2011 [57]	RCT (Between-subject design)	22 (men = 14, women = 8)	11	11	Mixed	22.7 ± 3.8	Recreationally active	Not reported	Hamstring Gluteal muscles	SLR angle
Mayorga-Vega et al. 2014 [60]	RCT (Between-subject design)	45 (men = 26, women = 19)	22 (men = 14, women = 8)	23 (men = 12, women = 11)	Mixed	IG 10.9 ± 0.3 CG10.9 ± 0.3	Mixed Extracurricular sports ≥ 2 × /week: 19/45 (42%)	Not reported	Hamstring	The classic sit-and-reach test
Mayorga-Vega et al. 2014 [64]	RCT (Between-subject design)	45 (men = 24, women = 21)	22 (men = 12, women = 10)	23 (men = 12, women = 11)	Mixed	IG 9.91 ± 0.29 CG 9.87 ± 0.34	Mixed Organized sports ≥ 2 × /week: IG 9 (41%), CG 9 (39%)	Not reported	Hamstring	Sit-and-reach test
Mayorga-Vega et al. 2015 [63]	RCT (Between-subject design)	180 (men = 94, women = 86)	IG1 52 (men = 27, women = 25) IG2 53 (men = 26, women = 27)	CG 58 (men = 31, women = 27)	Mixed	IG1 12.7 ± 0.7 IG2 12.7 ± 0.7 CG 12.6 ± 0.6	Mixed Extracurricular sports participation: IG (1 × /week) 37/53 (70%); IG (2 × /week) 35/52 (67%); CG 37/58 (64%)	Not reported	Hamstring	Classic sit-and-reach test
Mayorga-Vega et al. 2016 [58]	RCT (Between-subject design)	150 (men = 70, women = 80)	IG1 44 (men = 22, women = 22) IG2 51 (men = 23, women = 28)	CG 45 (men = 21, women = 24)	Mixed	IG1 8.5 ± 0.8 IG 8.4 ± 0.8 CG 8.4 ± 0.6	Mixed Extracurricular physical activity: some participants (children)	Mixed	Hamstring	The classic sit-and-reach test
Mayorga-Vega et al. 2017 [59]	RCT (Between-subject design)	37 (men = 18, women = 19)	19 (men = 8, women = 11)	18 (men = 6, women = 12)	Mixed	Not reported	Mixed Extracurricular sports participation: 17/37 (46%)	Not reported	Hamstring	The classic sit-and-reach test
Medina et al. 2007 [80]	RCT (Between-subject design)	62	IG1 25 IG2 20	18	Mixed	IG 10.3 ± 0.5 IG 10.5 ± 0.6 CG 10.3 ± 0.3	Not reported	Not reported	Hamstring	SLR angle
Melo et al. 2021 [89]	RCT (Between-subject design)	42	IG1 11 IG2 10 IG3 10	CG 10	Male	IG124.7 ± 4.8 IG224.7 ± 4.8 IG3 22.8 ± 2.1 CG 23.8 ± 4.1	Competitive athlete	Hamstring tightness (+)	Hamstring	Active knee extension test Passive knee extension test
Merino-Marban et al. 2015 [61]	RCT (Between-subject design)	45 (men = 26, women = 19)	23 (men = 12, women = 11)	22 (men = 14, women = 8)	Mixed	IG 5.91 ± 0.29 CG 5.91 ± 0.29	Mixed Extracurricular sports (≥ 2 × /week): IG 5 (22%), CG 7 (32%)	Not reported	Hamstring	Sit and reach test
Moltubakk et al. 2021 [65]	RCT (Contralateral extremity in within-subject design)	30	25		Mixed	22 ± 1.6	Recreationally active	Not reported	Ankle plantar flexors	Maximum ankle dorsiflexion angle
Muyor et al. 2012 [66]	RCT (Between-subject design)	58	27	31	Female	IG 44.85 ± 9.63 CG 43.56 ± 8.56	Not reported No participation in organized or non-organized physical exercise	Not reported	Hamstring	SLR angle Toe-touch test
Nakamura et al. 2012 [67]	RCT (Between-subject design)	18	9	9	Male	IG 21.1 ± 2.3 CG 21.8 ± 0.8	Not reported	Not reported	Ankle plantar flexors	Maximum knee flexion angle (prone)
Nakamura et al. 2021 [68]	RCT (Between-subject design)	40	13	13	Male	IG 21.4 ± 1 CG 21.9 ± 1.3	Not reported	Not reported	Ankle plantar flexors	Maximum ankle dorsiflexion angle
Nelson and Bandy 2004 [70]	RCT (Between-subject design)	81	21	24	Male	IG 16.29 ± 0.86 CG 16.24 ± 1.14	Not reported	Hamstring tightness (+)	Hamstring	Passive knee extension test
Nelson et al. 2012 [69]	RCT (Between-subject design)	25 (men = 12, women = 13)	13 (men = 6, women = 7)	12 (men = 6, women = 6)	Mixed	IG (male) 24 ± 3 IG (female) 25 ± 7 CG (male) 22 ± 2 CG (female) 22 ± 1	Not reported Physically inactive or minimally recreationally active	Not reported	Ankle plantar flexors	Maximum ankle dorsiflexion angle
Panidi et al. 2021 [71]	RCT (contralateral extremity in within-subject design)	26	21		Female	13.5 ± 1.4	Athlete	Not reported	Ankle plantar flexors	Maximum ankle dorsiflexion angle during the wall push stretch
Peixinho et al. 2016 [90]	RCT (Between-subject design)	18	8	8	Male	18.73 ± 0.36	Physically active	Not reported	Ankle plantar flexors	Maximum ankle dorsiflexion angle
Peixinho et al. 2021 [72]	RCT (Between-subject design)	20	12	8	Male	18.94 ± 0.45	Physically active	Not reported	Ankle plantar flexors	Maximum knee flexion angle
Peres et al. 2002 [74]	RCT (Between-subject design)	60	11	8	Mixed	22.5 ± 2	Not reported	Not reported	Ankle plantar flexors	Ankle dorsiflexion angle during traction (prone; pulley; 1/3 body weight load)
Reid and McNair 2004 [75]	RCT (Between-subject design)	43	23	20	Male	IG 15.83 ± 1.11 CG 15.67 ± 0.86	Not reported	Not reported	Hamstring	Maximum knee extension angle at 115° hip flexion
Rosenfeldt et al. 2024 [77]	RCT (Between-subject design)	18 (men = 5, women = 13)	6 (men = 1, women = 5)	6 (men = 1, women = 5)	Mixed	IG 24.1 ± 4.0 CG 24.8 ± 2.6	Physically active	Not reported	Hamstring	Sit and reach test
Ross 2007 [78]	RCT (contralateral extremity in within-subject design)	13 (men = 8, women = 5)	13		Mixed	20.3 ± 1.5	Physical activity ≥ 5 days/week (including organized sports)	Hamstring tightness (+)	Hamstring	Active knee extension test
Sainz de Baranda et al. 2006 [79]	RCT (Between-subject design)	63 (men = 31, women = 32)	45 (men = 22, women = 23)	18 (men = 9, women = 9)	Mixed	IG 10.5 ± 0.5 CG 10.3 ± 0.3	Physically active	Not reported	Hamstring	Toe touch test
Souza et al. 2017 [81]	RCT (Between-subject design)	40	19	19	Male	18.3 ± 0.5	Not reported	Hamstring tightness (-)	Hamstring	Passive knee extension test
Warneke et al. 2023 [91]	RCT (Between-subject design)	69 (men = 30, women = 39)	23 (men = 14, women = 10)	22 (men = 12, women = 11)	Mixed	IG 27.4 ± 3.1 CG 27.9 ± 6.1	Recreationally active	Not reported	Ankle plantar flexors	Knee-to-wall test Maximum ankle dorsiflexion angle
Warneke et al. 2023 [92]	RCT (Between-subject design)	80 (men = 45, women = 35)	IG1 20 (men = 11, women = 9) IG2 20 (men = 10, women = 10) IG3 20 (men = 12, women = 8)	20 (men = 12, women = 10)	Mixed	IG1 25.5 ± 5.5 IG2 26.7 ± 2.5 IG3 24.9 ± 2.9 CG26.1 ± 3.3	Athlete	Not reported	Ankle plantar flexors	Knee to wall test Maximum knee flexion angle
Warneke et al. 2023 [91]	RCT (Between-subject design)	45 (men = 28, women = 17)	21	22 (men = 13, women = 9)	Mixed	IG 28.2 ± 2.3 CG 26.8 ± 3.6	Recreationally strength-trained	Not reported	Ankle plantar flexors	knee-to-wall test Maximum knee flexion angle
Webright et al. 1997 [93]	RCT (Between-subject design)	40 (men = 22, women = 18)	15	14	Mixed	IG 21.2 ± 3.65 CG 22.1 ± 2.5	Not reported	Hamstring tightness (+)	Hamstring	Acive knee extension test
Yıldırım et al. 2016 [83]	RCT (Between-subject design)	67 (men = 44, women = 23)	10	14	Mixed	IG 21.4 ± 0.8 CG 21.4 ± 1.8	Not reported	Hamstring tightness (+)	Hamstring	SLR angle
Youdas et al. 2003 [85]	RCT (Between-subject design)	101 (men = 38, women = 63)	IG1 22 (men = 9, women = 13) IG2 22 (men = 8, women = 14) IG3 21 (men = 9, women = 12)	CG 24 (men = 9, women = 15)	Mixed	IG1 38.3 9.7 IG2 35.4 ± 11.0 IG3 36.7 ± 11.4 CG 36 ± 12	Mixed Strenuous exercise or hard physical labor ≥ 1 × /week: women: 48% (26/54), men: 80% (28/35)	Not reported	Ankle plantar flexors	Maximum ankle dorsiflexion angle
Yuktasir and Kaya 2009 [86]	RCT (Between-subject design)	28	10	9	Male	21.82 ± 1.9	Not reported	Not reported	Hamstring	Passive knee extension test
Zaidi et al. 2023 [87]	RCT (Between-subject design)	30	10	10	Male	IG 59 ± 3.83 CG 58.7 ± 3.02	Not reported	Not reported	Hamstring	Active knee extension test Chair Sit-and-Reach Test

RCT, randomized controlled trial; IG, intervention group; CG, control group; SLR, straight leg raising

^a^The total sample size includes participants who dropped out and participants in intervention conditions other than static stretching

### Risk of Bias Assessment and Methodological Quality

**Fig. 2:**
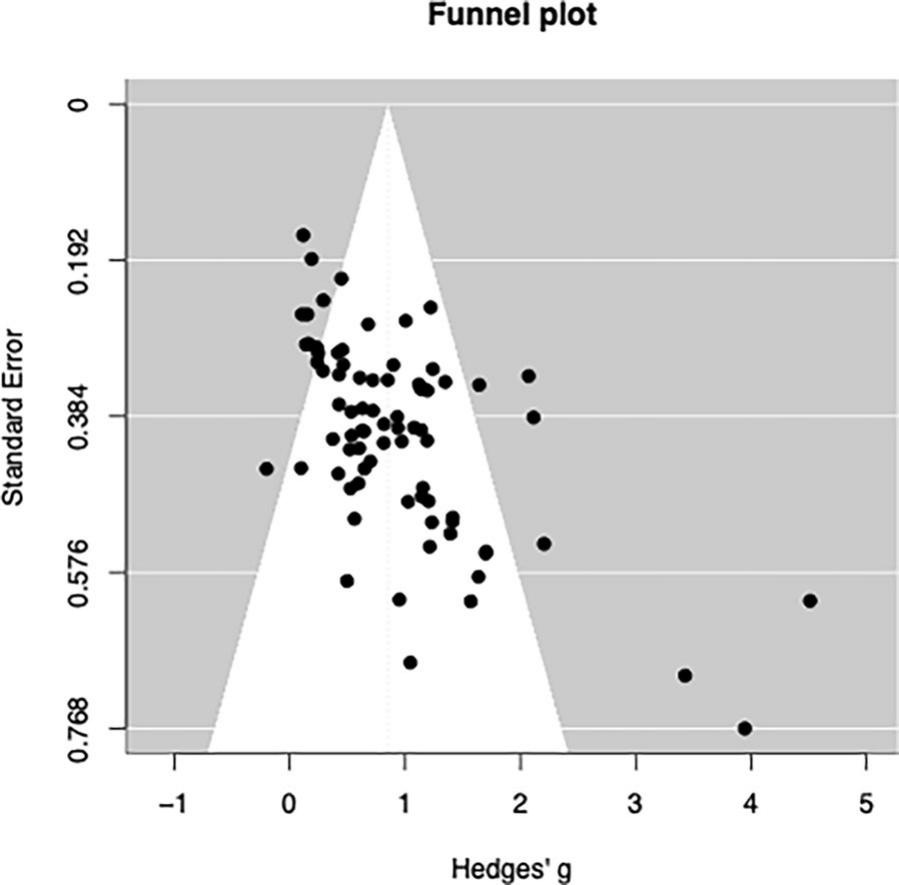
Funnel plot: relationship between effect sizes and standard errors for chronic static stretching effects on flexibility

Figure 2 presents a funnel plot of the studies included in the meta-analysis. Egger’s regression test indicated evidence of potential small-study effects (*p* < 0.001). Consequently, a trim-and-fill analysis was performed to assess the potential influence of small-study effects on the pooled effect estimate. No studies were imputed as potentially missing, and the adjusted pooled estimate remained unchanged. The corresponding funnel plot is presented in Additional file 1: Fig. S1. Between-study heterogeneity was substantial (τ^2^ = 0.249; I^2^ = 66.1%). The methodological quality of the included studies, assessed using the PEDro scale, is summarized in Table 2. The inter-rater agreement for the PEDro ratings was high, with a concordance rate of 95.51%. The mean PEDro score was 6.15 ± 0.90 (range 4–8), indicating overall moderate-to-good methodological quality [96].

**Table 2:** Risk of Bias assessment

Study	Random allocation	Concealed allocation	Baseline comparability	Blind participants	Blind therapists	Blind assessors	Adequate follow-up	Intention-to-treat analysis	Between-group comparisons	Point estimates and variability	Eligibility criteria^a^	Total Score
Akagi and Takahashi 2014 [17]	Yes	No	Yes	No	No	No	Yes	Yes	Yes	Yes	Yes	6
Andrade et al. 2020 [18]	Yes	Yes	Yes	Yes	No	No	Yes	No	Yes	Yes	Yes	7
Aquino et al. 2010 [29]	Yes	No	Yes	Yes	No	No	Yes	Yes	Yes	Yes	Yes	7
Ayala et al. 2010 [51]	Yes	No	Yes	Yes	No	No	Yes	Yes	Yes	Yes	Yes	7
Ayala et al. 2010 [51]	Yes	No	Yes	Yes	No	No	Yes	Yes	Yes	Yes	Yes	7
Ayala et al. 2013 [40]	Yes	Yes	Yes	Yes	No	No	Yes	No	Yes	Yes	Yes	7
Bandy and Irion 1994 [94]	Yes	No	Yes	Yes	No	No	Yes	No	Yes	Yes	Yes	6
Bandy et al. 1997 [84]	Yes	No	Yes	No	No	No	Yes	No	Yes	Yes	Yes	5
Bandy et al. 1998 [73]	Yes	No	Yes	Yes	No	No	Yes	Yes	Yes	Yes	Yes	7
Barbosa et al. 2018 [95]	Yes	Yes	Yes	Yes	No	No	No	No	Yes	Yes	Yes	6
Bazett-Jones et al. 2008 [19]	Yes	No	Yes	Yes	No	No	Yes	Yes	Yes	Yes	No	7
Ben and Harvey 2010 [20]	Yes	Yes	Yes	Yes	No	No	Yes	Yes	Yes	Yes	Yes	8
Blazevich et al. 2012 [21]	Yes	No	Yes	No	No	No	Yes	No	Yes	Yes	Yes	5
Borman et al. 2011 [22]	Yes	No	Yes	Yes	No	No	Yes	Yes	Yes	Yes	Yes	7
Caldwell et al. 2019 [23]	Yes	No	Yes	No	No	No	Yes	Yes	Yes	Yes	Yes	6
Castro et al. 2013 [33]	Yes	Yes	Yes	No	No	No	No	No	Yes	Yes	Yes	5
Chan et al. 2001 [24]	Yes	No	No	No	No	No	Yes	Yes	Yes	Yes	Yes	5
Chaudhuri and Kiba 2018 [25]	Yes	No	Yes	No	No	No	Yes	Yes	Yes	Yes	Yes	6
Christiansen 2008 [26]	Yes	Yes	Yes	Yes	No	No	Yes	No	Yes	Yes	Yes	7
Cini et al. 2020 [27]	Yes	No	Yes	Yes	No	No	Yes	Yes	Yes	Yes	Yes	7
Cini et al. 2024 [28]	Yes	No	Yes	Yes	No	No	Yes	Yes	Yes	Yes	Yes	7
Cipriani et al. 2012 [30]	Yes	Yes	Yes	Yes	No	No	Yes	No	Yes	Yes	Yes	7
Covert et al. 2010 [31]	Yes	No	Yes	Yes	No	No	Yes	Yes	Yes	Yes	Yes	7
Dal Paz et al. 2018 [32]	Yes	No	Yes	No	No	No	No	No	Yes	Yes	Yes	4
Fernandez et al. 2016 [76]	Yes	No	No	No	No	No	Yes	Yes	Yes	Yes	No	5
Folpp et al. 2006 [35]	Yes	Yes	Yes	Yes	No	No	Yes	Yes	Yes	Yes	Yes	8
Gajdosik et al. 2005 [37]	Yes	Yes	Yes	No	No	No	Yes	Yes	Yes	Yes	Yes	7
Gajdosik et al. 2007 [36]	Yes	No	Yes	Yes	No	No	No	No	Yes	Yes	No	5
Gallo et al. 2015 [88]	Yes	No	Yes	No	No	No	No	No	Yes	Yes	Yes	4
Godges et al. 1993 [38]	Yes	No	Yes	No	No	No	Yes	Yes	Yes	Yes	Yes	6
Gribble et al. 1999 [39]	Yes	No	Yes	No	No	No	Yes	No	Yes	Yes	Yes	5
Gunaydin et al. 2020 [41]	Yes	No	Yes	No	No	No	Yes	Yes	Yes	Yes	Yes	6
Henderson et al. 2025 [42]	Yes	Yes	Yes	No	No	No	Yes	No	Yes	Yes	Yes	6
Ikeda and Tomoo 2021 [43]	Yes	No	Yes	No	No	No	Yes	No	Yes	Yes	Yes	5
Johanson et al. 2009 [44]	Yes	No	Yes	No	No	No	Yes	Yes	Yes	Yes	Yes	6
Johnson et al. 2014 [45]	Yes	Yes	Yes	Yes	No	No	No	No	Yes	Yes	Yes	6
Junpei et al. 2023 [46]	Yes	No	Yes	No	No	No	Yes	Yes	Yes	Yes	Yes	6
Kim et al. 2017 [47]	Yes	Yes	Yes	Yes	No	No	Yes	Yes	Yes	Yes	Yes	8
Knight et al. 2001 [48]	Yes	No	Yes	No	No	No	Yes	Yes	Yes	Yes	Yes	6
Kokkonen et al. 2007 [49]	Yes	No	Yes	No	No	No	Yes	No	Yes	Yes	Yes	5
Konrad and Tilp 2014 [50]	Yes	No	Yes	No	No	No	Yes	No	Yes	Yes	Yes	5
Laudner et al. 2016 [52]	Yes	No	Yes	Yes	No	No	Yes	Yes	Yes	Yes	Yes	7
Li et al. 1996 [53]	Yes	No	Yes	No	No	No	Yes	Yes	Yes	Yes	Yes	6
Lima et al. 2015 [34]	Yes	No	Yes	Yes	No	No	Yes	No	Yes	Yes	Yes	6
Longo et al. 2021 [54]	Yes	Yes	Yes	No	No	No	Yes	No	Yes	Yes	Yes	6
Mahieu et al. 2007 [55]	Yes	Yes	Yes	Yes	No	No	Yes	No	Yes	Yes	Yes	7
Maras et al. 2024 [56]	Yes	No	Yes	Yes	No	No	Yes	Yes	Yes	Yes	Yes	7
Marshall et al. 2011 [57]	Yes	No	Yes	No	No	No	Yes	Yes	Yes	Yes	Yes	6
Mayorga-Vega et al. 2014 [60]	Yes	No	Yes	No	No	No	Yes	Yes	Yes	Yes	Yes	6
Mayorga-Vega et al. 2014 [64]	Yes	No	Yes	No	No	No	Yes	Yes	Yes	Yes	Yes	6
Mayorga-Vega et al. 2015 [63]	Yes	No	Yes	No	No	No	Yes	No	Yes	Yes	Yes	5
Mayorga-Vega et al. 2016 [58]	Yes	No	Yes	No	No	No	Yes	No	Yes	Yes	Yes	5
Mayorga-Vega et al. 2017 [59]	Yes	No	Yes	No	No	No	Yes	Yes	Yes	Yes	Yes	6
Medina et al. 2007 [80]	Yes	No	Yes	Yes	No	No	Yes	Yes	Yes	Yes	No	7
Melo et al. 2021 [89]	Yes	Yes	Yes	Yes	No	No	Yes	No	Yes	Yes	Yes	7
Merino-Marban et al. 2015 [61]	Yes	No	Yes	No	No	No	Yes	Yes	Yes	Yes	Yes	6
Moltubakk et al. 2021 [65]	Yes	No	Yes	Yes	No	No	Yes	No	Yes	Yes	Yes	6
Muyor et al. 2012 [66]	Yes	No	Yes	No	No	No	Yes	Yes	Yes	Yes	Yes	6
Nakamura et al. 2012 [67]	Yes	No	Yes	No	No	No	Yes	Yes	Yes	Yes	Yes	6
Nakamura et al. 2021 [68]	Yes	No	Yes	No	No	No	Yes	Yes	Yes	Yes	Yes	6
Nelson and Bandy 2004 [70]	Yes	No	Yes	No	No	No	Yes	No	Yes	Yes	Yes	5
Nelson et al. 2012 [69]	Yes	No	Yes	No	No	No	Yes	Yes	Yes	Yes	Yes	6
Panidi et al. 2021 [71]	Yes	No	Yes	Yes	No	No	Yes	No	Yes	Yes	Yes	6
Peixinho et al. 2016 [90]	Yes	No	Yes	No	No	No	Yes	Yes	Yes	Yes	No	6
Peixinho et al. 2021 [72]	Yes	No	Yes	Yes	No	No	Yes	Yes	Yes	Yes	Yes	7
Peres et al. 2002 [74]	Yes	No	Yes	No	No	No	Yes	Yes	Yes	Yes	Yes	6
Reid and McNair 2004 [75]	Yes	No	Yes	Yes	No	No	Yes	Yes	Yes	Yes	Yes	7
Rosenfeldt et al. 2024 [77]	Yes	Yes	Yes	No	No	No	Yes	Yes	Yes	Yes	Yes	7
Ross 2007 [78]	Yes	No	Yes	Yes	No	No	Yes	Yes	Yes	Yes	Yes	7
Sainz de Baranda et al. 2006 [79]	Yes	No	Yes	Yes	No	No	Yes	Yes	Yes	Yes	No	7
Souza et al. 2017 [81]	Yes	No	Yes	No	No	No	Yes	No	Yes	Yes	Yes	5
Warneke et al. 2023 [91]	Yes	No	Yes	No	No	No	Yes	Yes	Yes	Yes	Yes	6
Warneke et al. 2023 [92]	Yes	No	Yes	No	No	No	Yes	Yes	Yes	Yes	Yes	6
Warneke et al. 2023 [91]	Yes	No	Yes	No	No	No	Yes	No	Yes	Yes	Yes	5
Webright et al. 1997 [93]	Yes	No	Yes	No	No	No	Yes	Yes	Yes	Yes	Yes	6
Yıldırım et al. 2016 [83]	Yes	No	Yes	Yes	No	No	No	No	Yes	Yes	Yes	5
Youdas et al. 2003 [85]	Yes	No	Yes	No	No	No	Yes	No	Yes	Yes	Yes	5
Yuktasir and Kaya 2009 [86]	Yes	No	Yes	Yes	No	No	Yes	Yes	Yes	Yes	Yes	7
Zaidi et al. 2023 [87]	Yes	Yes	Yes	Yes	No	No	Yes	Yes	Yes	Yes	Yes	8

^a^Criterion of eligibility criteria was not counted in the total score

### Chronic Stretching Effects

**Fig. 3:**
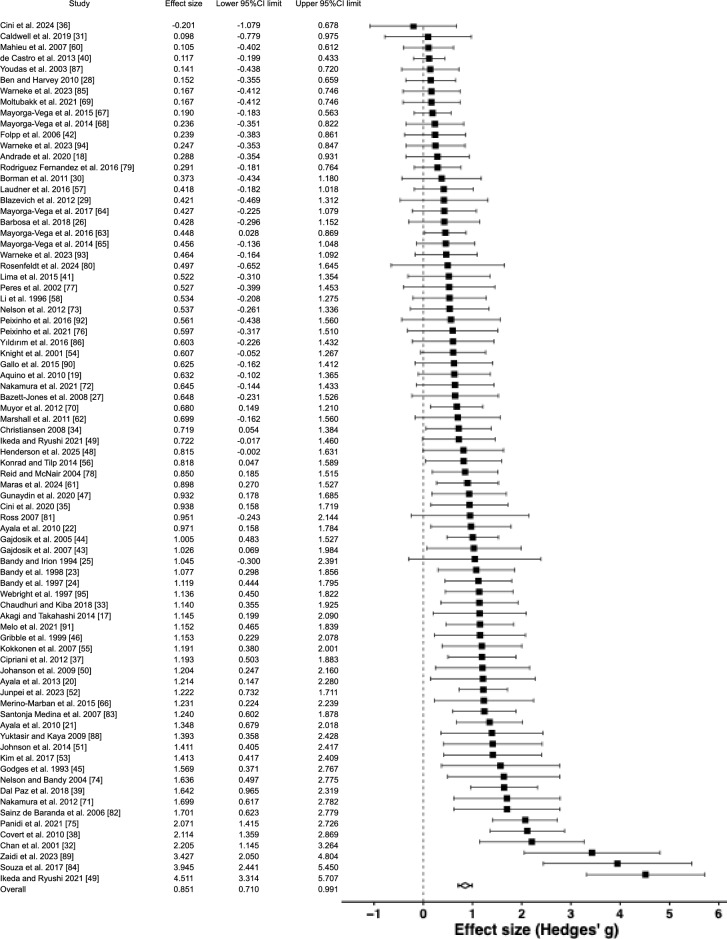
Forest plot summarizing the effects of chronic static stretching on flexibility in all included studies

Overall, chronic static stretching demonstrated a moderate and significant effect on flexibility improvement (Hedges’ g = 0.851, 95% confidence interval [CI] 0.710–0.991, standard error [SE] = 0.0718, Z = 11.845, *p* < 0.001) (Fig. 3). The test for homogeneity indicated significant between-study heterogeneity (Q = 227.644, df = 78, *p* < 0.001), with substantial heterogeneity (τ^2^ = 0.249, I^2^ = 66.1%). The subgroup analyses (Table 3) showed no significant moderating effects for age (children/adolescents vs young adults vs middle-aged adults vs older adults, *p* = 0.853); sex (male vs female, *p* = 0.990); training status (athlete/trained vs recreationally active vs sedentary, *p* = 0.221); baseline flexibility (with tightness vs without tightness/not reported, *p* = 0.260); or target muscle group (knee flexors vs knee extensors vs ankle plantar flexors, *p* = 0.100).

**Table 3:** Subgroup effect estimates and statistics

Subgroup		Number of studies	Hedges’ g (95% CI)	Z-value	*p* value	I^2^	Q statistics
Age	Children/adolescents	13	0.838 (0.448–1.227)	4.212	< 0.001	85.172	Q = 0.786; df = 3; *p* = 0.853
	Young	59	0.824 (0.674–0.974)	10.747	< 0.001	54.139	
	Middle-aged	3	1.572 (-0.567–3.712)	1.440	0.150	94.657	
	Older	4	0.894 (0.480–1.309)	4.228	< 0.001	0.0002	
Sex	Male	20	1.151 (0.727–1.575)	5.320	< 0.001	84.784	Q = 0.000; df = 1; *p* = 0.990
	Female	9	1.105 (0.710–1.500)	5.486	< 0.001	42.661	
Training status	Athlete and trained	9	0.751 (0.317–1.184)	3.393	< 0.001	75.720	Q = 3.021; df = 2; *p* = 0.221
	Recreational and active	19	0.544 (0.338–0.750)	5.177	< 0.001	34.408	
	Sedentary	3	1.279 (0.590–1.967)	3.637	< 0.001	0.000	
Baseline flexibility	With tightness	22	0.953 (0.760–1.145)	9.714	< 0.001	22.054	Q = 1.270; df = 1; *p* = 0.260
	Without tightness and not reported	57	0.818 (0.636–1.001)	8.771	< 0.001	74.110	
Muscle group	Knee flexor	47	0.995 (0.789–1.201)	9.456	< 0.001	73.724	Q = 4.608; df = 2; *p* = 0.100
	Knee extensor	6	0.550 (0.199–0.902)	3.068	0.002	0.0002	
	Ankle plantar flexor	22	0.656 (0.437–0.875)	5.870	< 0.001	48.090	

95% CI, 95% confidence interval; df, degree of freedom

### Sensitivity Analyses

Sensitivity analyses were performed to determine whether the pooled estimate was materially influenced by study design or unusually large effects. First, excluding the five studies that used a within-subject contralateral-limb control design did not meaningfully change the pooled effect, which remained statistically significant (Hedges’ g = 0.845, 95% CI = 0.702–0.988, *p* < 0.001; τ^2^ = 0.235; I^2^ = 64.6%). Second, Zaidi et al. [87] reported a markedly larger effect than the remaining studies and was considered a likely contributor to the observed small-study effects and heterogeneity. Excluding this study with extreme effect size, based on the predefined interpretation threshold, did not change the overall conclusion, although the pooled effect size was attenuated (Hedges’ g = 0.805, 95% CI 0.679–0.931, *p* < 0.001) and heterogeneity was reduced (τ^2^ = 0.170; I^2^ = 57.3%). Egger’s regression test remained statistically significant even after excluding these studies (*p* < 0.001), indicating that small-study effects persisted and were not fully explained by the extreme effect sizes alone.

## Discussion

This systematic review and meta-analysis demonstrated that chronic static stretching produces significant and moderate improvements in flexibility. The results indicated that age, sex, baseline flexibility, training status, and target muscle group did not significantly moderate the chronic flexibility adaptations in lower-limb muscles. These findings suggest that static stretching is generally effective in enhancing lower-limb flexibility across diverse healthy individuals and target muscle groups.

In this systematic review and meta-analysis, the pooled analysis showed that chronic static stretching resulted in a significant, moderate improvement in flexibility, consistent with the results of the previous meta-analyses of chronic stretching interventions [5–8]. Unlike earlier reviews that included non-randomized designs, combined upper and lower limb outcomes, and participants with heterogeneous health conditions, we strengthened the internal validity by focusing on RCTs of lower limb muscles in healthy individuals. Consequently, the observed effect size, with a relatively narrow CI, provides robust evidence supporting the chronic benefits of static stretching. These findings have practical implications for sports performance, rehabilitation, and fall prevention, particularly in contexts where lower limb flexibility is a relevant and modifiable outcome.

The subgroup analysis did not show a significant moderator effect of target muscle group (knee extensors vs knee flexors vs plantar flexors) (*p* = 0.100). Significant improvements in flexibility were observed across all examined muscle groups. This finding is generally consistent with those of previous systematic reviews and meta-analyses by Ingram et al. [5] and Konrad et al. [6], who reported that chronic static stretching improves flexibility across different muscle groups. Therefore, the present results suggest that chronic static stretching can improve flexibility regardless of the target lower-limb muscle group. Although the pooled estimate was numerically largest for the knee flexors, the difference among muscle groups was not significant. Accordingly, this apparent difference should be interpreted cautiously and may reflect methodological differences in flexibility assessment, stretching techniques, or intervention protocols rather than a true muscle group–specific adaptation. Collectively, these findings indicate that meaningful improvements in flexibility can be achieved across the knee extensors, knee flexors, and plantar flexors.

The subgroup analyses revealed that stretching-induced improvements in flexibility were not moderated by age (*p* = 0.853). These findings are consistent with those reported by Ingram et al. [5], who observed no difference in the chronic stretching effect between participants aged < 65 years and those aged ≥ 65 years. By contrast, Arntz et al. [7] conducted a meta-regression analysis of stretching-induced changes in muscle strength and power. Their analysis suggests that age may serve as a moderating factor in strength adaptations. In the present study, a detailed subgroup analysis across four age categories (< 18 years, 18–39 years, 40–64 years, and ≥ 65 years) consistently demonstrated moderate effect sizes in all groups. ROM and the mechanical properties of the muscle–tendon unit change with age [97, 98], the chronic effects of static stretching may be influenced by these age-related alterations. However, a meta-analysis examining the acute effects of static stretching on muscle–tendon unit mechanical properties between younger and older adults reported no significant age-related differences [99]. With respect to chronic effects, no studies directly comparing age groups were identified among those included in the present review. Nevertheless, the findings of the previous meta-analysis [99] provide partial support for the subgroup findings observed herein. Collectively, these data suggest that although baseline and absolute ROM may vary across age groups, the magnitude of ROM improvement following chronic static stretching, expressed as standardized effect sizes rather than absolute ROM values or percentage changes, may not differ substantially by age.

Our subgroup analysis also indicated that the chronic effects of stretching on flexibility were not significantly moderated by sex (*p* = 0.990), consistent with the results of previous study [5]. By contrast, Arntz et al. reported that intervention groups with a higher proportion of women demonstrated greater gains in muscle strength. Similarly, Konrad et al. [6] found that stretching programs involving only female participants—incorporating modalities such as dynamic, ballistic, and PNF stretching—produced larger improvements in flexibility. Collectively, these findings suggest that women may exhibit greater adaptive responses when the outcome involves muscle strength or when stretching protocols require substantial muscular contractions.

Regarding the participant’s training status, the subgroup analysis revealed no significant differences in flexibility adaptations to static stretching (athletes and trained vs recreationally active vs sedentary) (*p* = 0.221). Konrad et al. [6] suggested that trained and untrained individuals may respond differently to training stimuli, such as concurrent resistance and endurance training [100], and hypothesized that training status could influence flexibility improvements. However, neither their findings nor those of the present study support this hypothesis. Moderate improvements in flexibility were observed consistently across all training status groups. These findings have practical implications, indicating that similar improvements in flexibility can be achieved by athletes, recreationally active individuals, and sedentary participants alike.

The subgroup analysis revealed no significant differences in baseline flexibility (*p* = 0.260). This finding contrasts with the interpretation of Ingram et al. [5], who suggested that individuals with greater baseline flexibility may exhibit smaller improvements as their ROM approaches the theoretical upper limit. However, this interpretation assumes that participants with excessive flexibility were represented in the subgroup analysis; thus, the result does not directly compare with those of subgroup analyses contrasting participants with tightness and those with normal flexibility. Notably, the study by Ayala et al. [40], which was included in both Ingram et al.’s meta-analysis [5] and the present study, reported consistent ROM increases over the 12-week intervention period, with comparable magnitudes observed in both the tight and normal hamstring groups. Collectively, the available evidence suggests that limited baseline flexibility is unlikely to constrain stretch-induced improvements. In the included studies, baseline flexibility was generally categorized as “tight,” “normal,” or “not reported,” whereas excessive flexibility was rarely defined. Future research should standardize criteria for tightness and explicitly include participants with flexibility levels approaching the theoretical upper limit (i.e., individuals with exceptionally high baseline flexibility) to better elucidate the role of baseline flexibility in static stretching adaptations.

This study has some limitations. First, substantial heterogeneity remained evident despite restricting the inclusion criteria to RCTs and lower limb muscles. The observed heterogeneity may reflect intervention-related factors that were not examined as moderators in the present review, including stretch volume, intensity, frequency, number of sets, session duration, and total intervention duration [4]. Because the present review focused primarily on individual characteristics and target muscle group and because these intervention-related variables were inconsistently reported across included studies, their potential contribution to heterogeneity could not be fully addressed. Second, this review was restricted to lower-limb muscles and joints; therefore, the findings may not be generalizable to upper-limb flexibility adaptations. Third, this review only included studies published in English, potentially excluding relevant literature published in other languages. However, previous evidence [101] suggests that restricting systematic reviews to English-language publications exerts only a minimal influence on pooled estimates and overall conclusions. Fourth, recent studies have evaluated the chronic effects of static stretching on muscle–tendon unit stiffness, muscle stiffness, and muscle architecture. As this review focused exclusively on flexibility outcomes, the findings should be interpreted with caution, as these measures reflect distinct physiological constructs. Finally, we did not perform a formal GRADE assessment of the certainty of evidence. Although such an assessment would further strengthen the interpretation of the findings, the present review was primarily designed to examine potential moderators of flexibility adaptations rather than to formulate clinical recommendations. In addition, the included studies showed substantial variability in stretching protocols, target muscle groups, outcome measures, and participant characteristics. Therefore, the findings should be interpreted cautiously, particularly in light of the substantial heterogeneity, potential small-study effects, small sample sizes in several studies, and methodological limitations inherent to stretching interventions, such as the difficulty of blinding participants and therapists.

## Conclusions

This systematic review and meta-analysis examined the chronic effects of static stretching on lower-limb flexibility, using evidence restricted to RCTs. Pooled results showed that chronic static stretching was associated with significant and moderate improvements in flexibility. However, substantial heterogeneity (τ^2^ = 0.249; I^2^ = 66.1%) and potential small-study effects were observed, warranting cautious interpretation of the findings. Subgroup analyses suggested that these adaptations were not meaningfully moderated by age, sex, baseline flexibility, training status, or target lower-limb muscle group. Collectively, the findings suggest that static stretching may improve lower-limb flexibility across diverse healthy individuals and target muscle groups, although the certainty and generalizability of these findings are limited by heterogeneity, potential small-study effects, and methodological variability among the included studies.

## Supplementary Information

**Additional file 1: Fig. S1**. Funnel plot based on trim-and-fill analysis. The trim-and-fill method did not impute any potentially missing studies; therefore, the adjusted pooled estimate remained unchanged.

## Data Availability

The datasets generated during this study (e.g., extracted data and risk-of-bias assessments) are available from the corresponding author upon reasonable request.
