# Supplementary figures and images for: Moderating Effects of Individual Characteristics and the Target Lower Limb Muscle Group on Flexibility Adaptations to Chronic Static Stretching in Healthy Individuals: A Systematic Review and Meta-Analysis of Randomized Controlled Trials
Source: Sports Med Open. 2026 Jul 11;12:95. doi: 10.1186/s40798-026-01066-1 (PMC13356130; doi:10.1186/s40798-026-01066-1)

## Slide 1
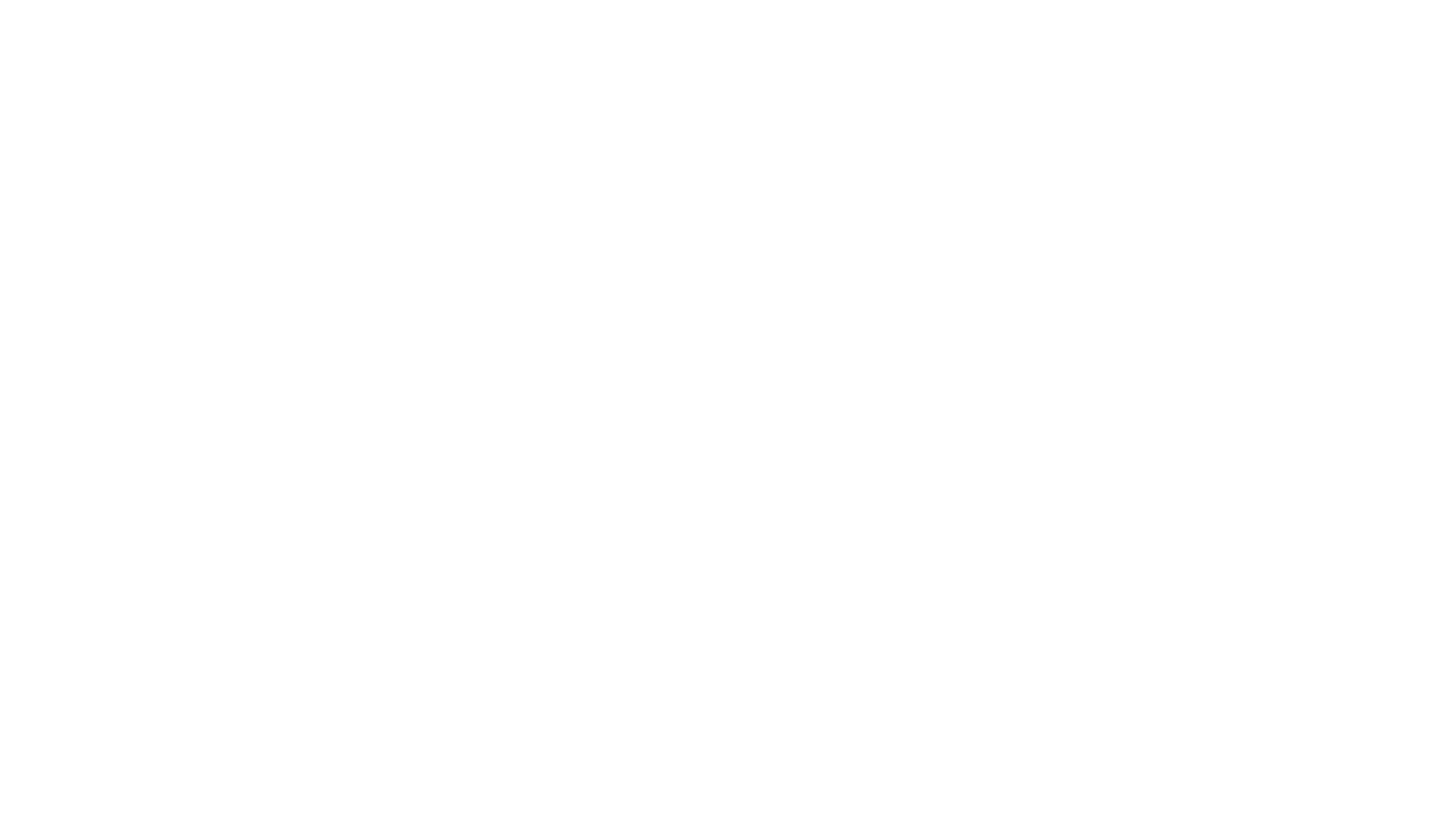

Supplement: Supplementary file 1 — Additional file 1: Fig. S1. Funnel plot based on trim-and-fill analysis. The trim-and-fill method did not impute any potentially missing studies; therefore, the adjusted pooled estimate remained unchanged. [file 40798_2026_1066_MOESM1_ESM.pptx]
